# Supplementary material for: Tropical forest carbon sequestration accelerated by nitrogen
Source: Nat Commun. 2026 Jan 13;17:55. doi: 10.1038/s41467-025-66825-2 (PMC12800027; doi:10.1038/s41467-025-66825-2)
Supplement: Supplementary file 1 — Supplementary Information [file 41467_2025_66825_MOESM1_ESM.pdf]

**Supplementary Information**

**for**

# Tropical forest carbon sequestration accelerated by nitrogen

**Authors:** Wenguang Tang[1,2], Jefferson S. Hall[3], Oliver L. Phillips[1], Roel J. W. Brienen[1], S. Joseph Wright[4], Michelle Y. Wong[4,5,6], Lars O. Hedin[7], Michiel van Breugel[4,8], Joseph B. Yavitt[9], Phillip M. Hannam[5], and Sarah A. Batterman[1,4,5*]

**Affiliations:**

[1]School of Geography, University of Leeds, Leeds, UK
[2]School of Geographical and Earth Sciences, University of Glasgow, Glasgow, UK
[3]ForestGEO, Smithsonian Tropical Research Institute, Ancón, Panamá, Panama
[4]Smithsonian Tropical Research Institute, Apartado 0843–03092, Balboa, Panama
[5]Cary Institute of Ecosystem Studies, Millbrook, NY, USA
[6]Department of Ecology and Evolutionary Biology, Yale University, New Haven, CT, USA
[7]Department of Ecology and Evolutionary Biology, Princeton University, Princeton, NJ, USA
[8]Department of Geography, National University of Singapore, Singapore, Singapore
[9]Department of Natural Resources and the Environment, Cornell University, Ithaca, NY, USA

*Corresponding author: Sarah Batterman. Email: battermans@caryinstitute.org

**Supplementary Information 1.**

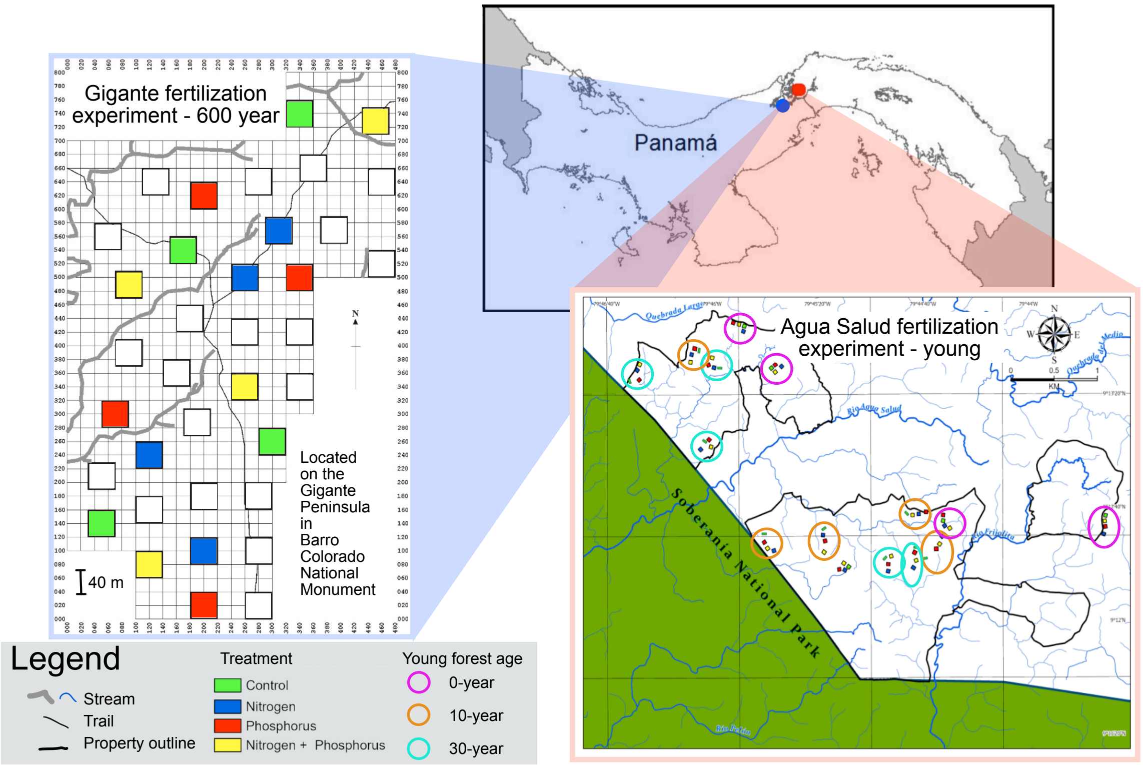


**Supplementary Fig. 1. Map of the location of the fertilization experiments and the layout of the nutrient addition plots.** The experiments were established in two nearby sites in central Panama, Agua Salud and Gigante. The Agua Salud experiment includes three forests: 0-year-old forest (purple circles), 10-year-old forest (orange circles), and 30-year-old forest (blue circles). Each forest had five nutrient fertilization blocks (replicates), and each block contained a control plot (green rectangle or square), a nitrogen added plot (blue square), a phosphorus added plot (red square), and a nitrogen plus phosphorus added plot (yellow square). The Gigante experiment had one age, the 600-year-old forest. White plots at Gigante were not included in this analysis. The Agua Salud fertilization experiment is situated within a ~15 square kilometer landscape and the Gigante experiment is situated within a ~0.4 square kilometer landscape.

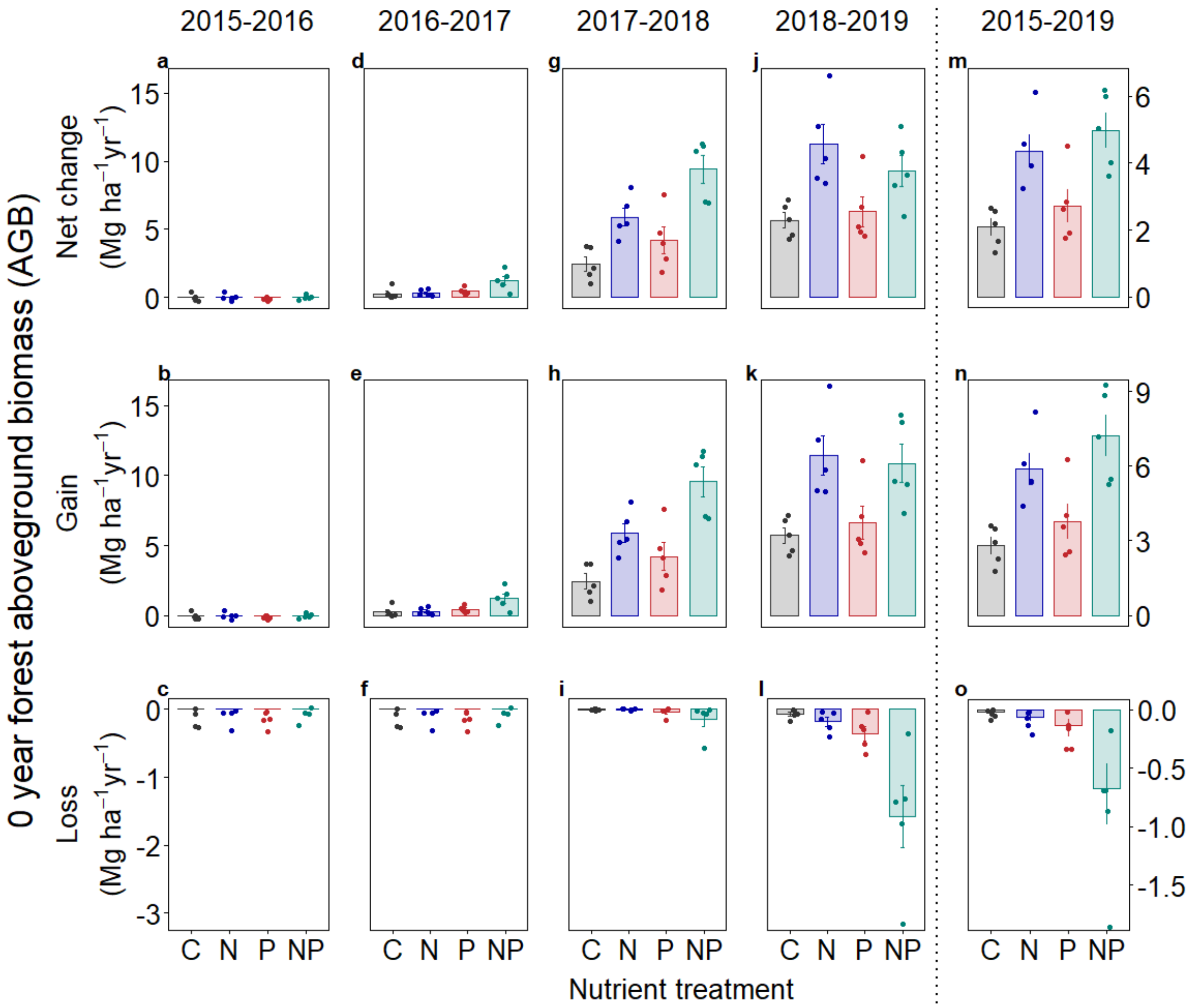


**Supplementary Fig. 2. The effect of nutrients on aboveground biomass net change, gain and loss in the 0-year forest for each annual census interval.** Each bar represents the mean +/- standard error of the mean (s.e.m.) and each point represents the data for individual plots. Aboveground biomass net change (a) and gain (b) in the interval 2015-2016 and biomass loss (c and f) in the intervals 2015-2016 and 2016-2017 are 0 Mg ha$^{-1}$ yr$^{-1}$.

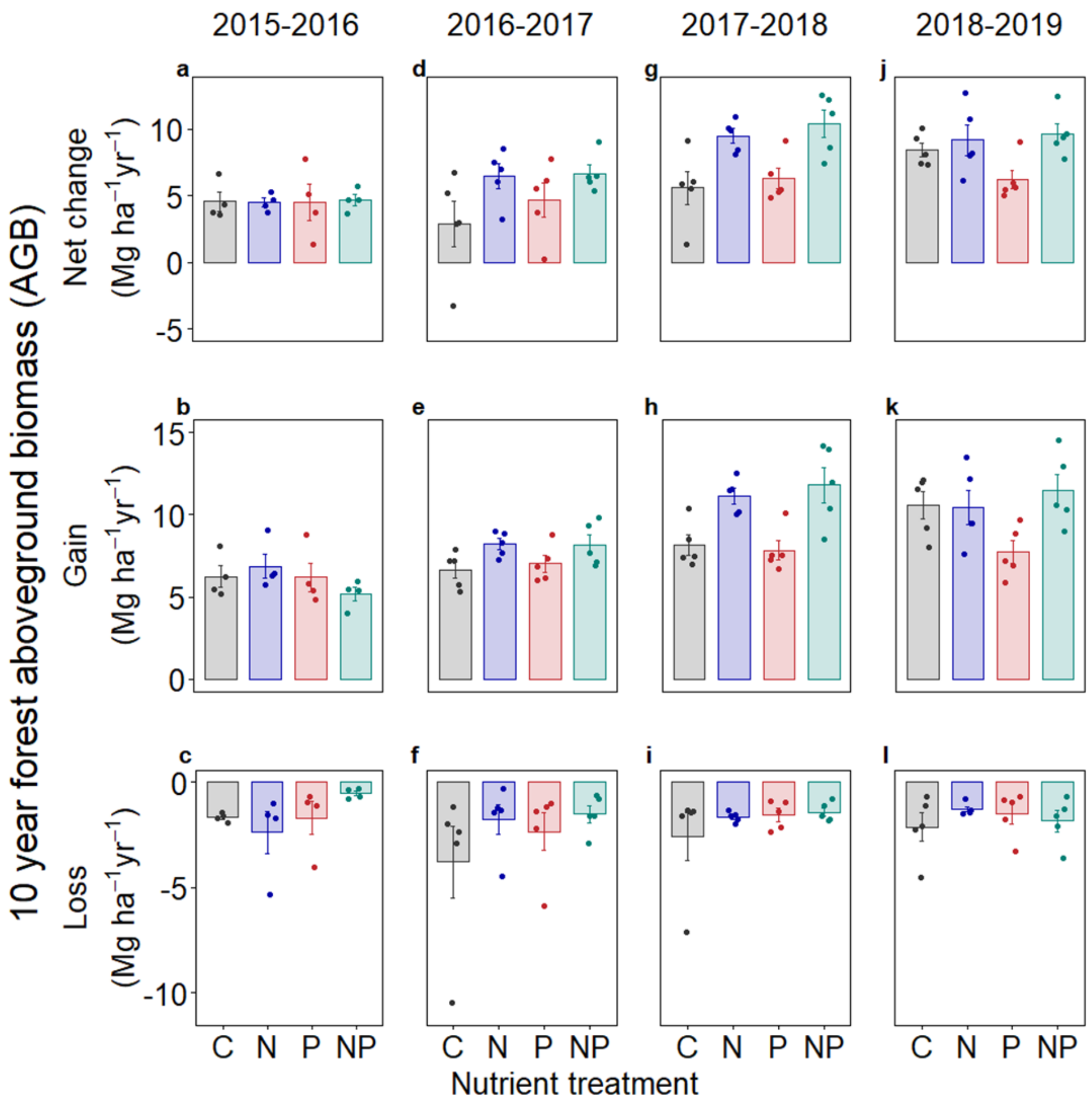


**Supplementary Fig. 3. The effect of nutrients on aboveground biomass net change, gain and loss in the 10-year forest for each annual census interval.** Each bar represents the mean +/- standard error of the mean (s.e.m.) and each point represents the data for individual plots.

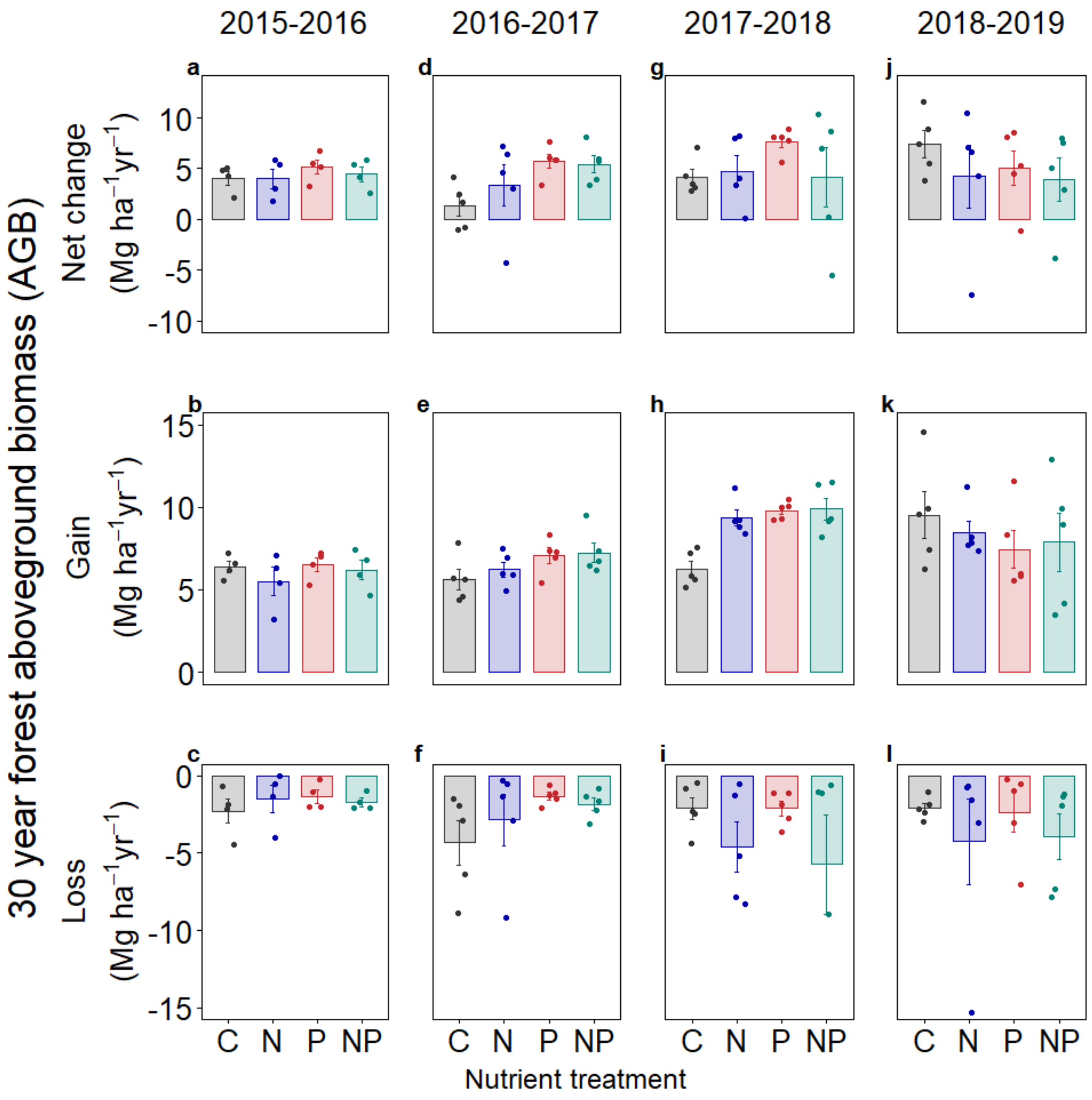


**Supplementary Fig. 4. The effect of nutrients on aboveground biomass net change, gain and loss in the 30-year forest in each annual interval.** Each bar represents the mean +/- standard error of the mean (s.e.m.) and each point represents the data for individual plots.

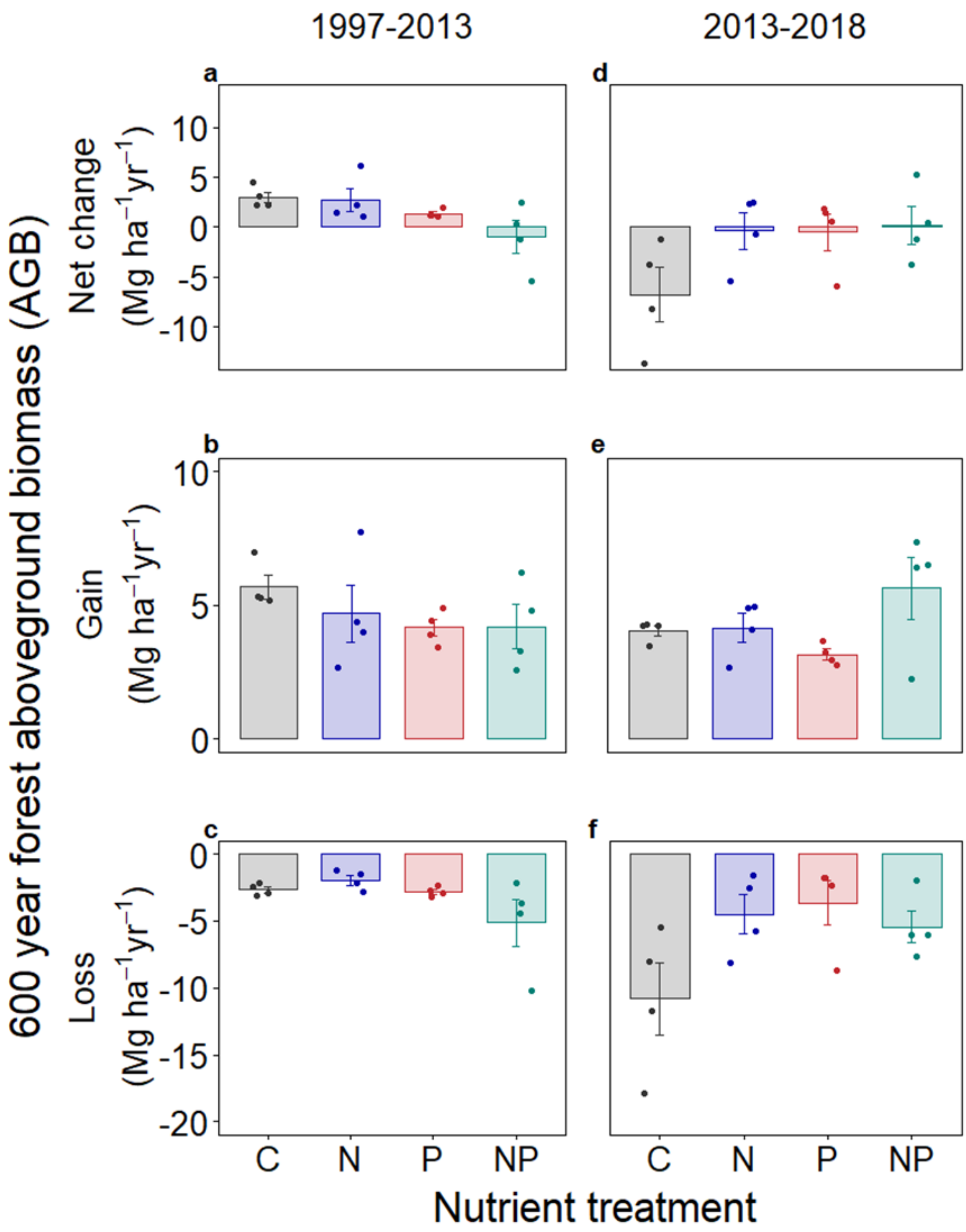


**Supplementary Fig. 5. The effect of nutrients on aboveground biomass net change, gain and loss in 600-year forest across census intervals.** Each bar represents the mean +/- standard error of the mean (s.e.m.) and each point represents the data for individual plots. Data for the 1997 to 2013 interval represent the mean of 1997-2003, 2003-2008, and 2008-2013.

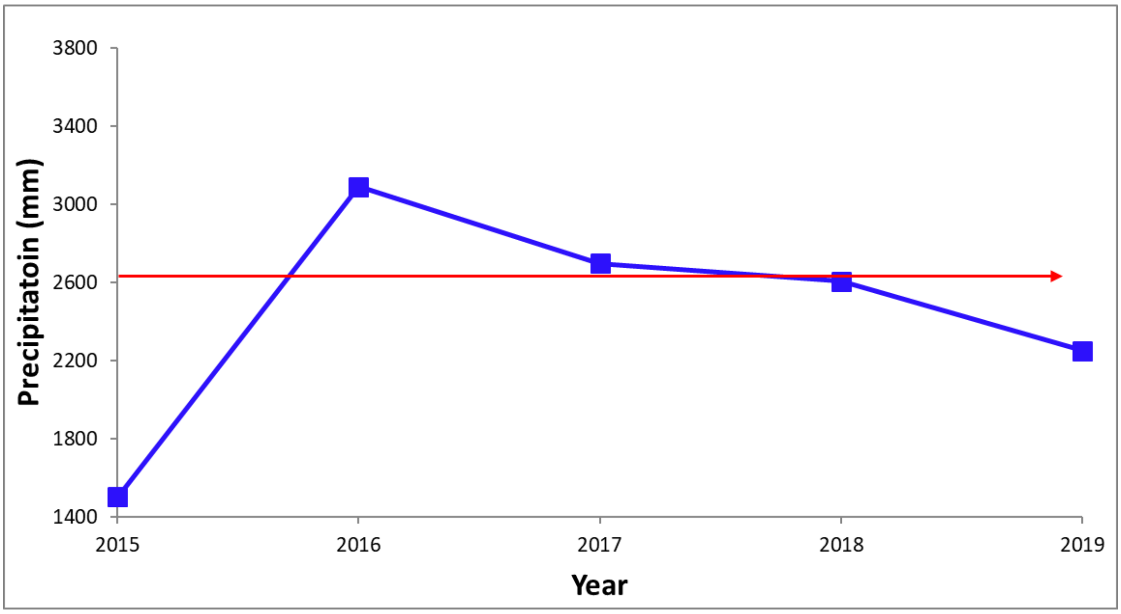


**Supplementary Fig. 6. Annual precipitation at Agua Salud during the experimental period.** The blue dots represent the annual precipitation during the experiment and the red line represents mean annual precipitation from 2009 to 2019.

**Supplementary Table 1. Soil properties recorded closest to 2015 for secondary forests at Agua Salud and for old-growth forest at Gigante.** Supplementary Table 1 summarizes soil properties for the 0-, 10-, and 30-year-old forests at Agua Salud and the mature forest at Gigante. Soils from both sites were analyzed in the Soil Biogeochemistry Lab at the Smithsonian Tropical Research Institute in Panama using identical methods. Soil pH was determined in both distilled water ($H_2O$) and calcium chloride ($CaCl_2$). Extractable nitrogen ($NH_4^+$ and $NO_3^-$) was determined with 2 M potassium chloride extract and colorimetry. Exchangeable potassium (K) was determined with 0.1 M $BaCl_2$ extract and quantified using inductively coupled plasma optical emission spectrometry[1]. Available phosphorus (P) was determined with exchange resins and molybdate colorimetry. Total carbon (C) and (N) were determined with a C-N analyzer. Total phosphorus was determined with nitric acid extraction and detection with ICP-OES.

At Agua Salud, soil properties were determined for the top 10 cm of soil for all plots prior to fertilization in 2015 and 2016 (n=20 plots per forest age). At Gigante, soil properties were determined repeatedly[2]. Values are for the top 10 cm of soil from the four control plots were collected closest to 2015 unless noted differently. Sand, silt, and clay fractions were determined for all 32 experimental plots in 2006[3]. Soil pH, extractable nutrient concentrations, and total C and N were determined in 2017[4]. Total phosphorus was determined in 2017 (see ref.[4]).

In the Supplementary Table 1, entries are means ± 1 stand error (s.e.m.). Sample size is five control plots for each forest age at Agua Salud and four control plots at Gigante. Asterisks (*) denote significant differences with age determined by ANOVA in R (4.0.2)[5]. The letters A, B and C denote significant differences between forest ages determined with *post hoc* contrasts (using emmeans function from the 'emmeans' package). Sample size is 60 plots for Agua Salud (pre-manipulation), 32 plots for sand/silt/clay for Gigante and four control plots for all other Gigante variables. See the statistical analysis and data in ref.[4].

| Forest age | Sand (%) | Silt (%) | Clay (%) | pH ($H_2O$) | pH ($CaCl_2$) | $NH_4^+$ * (mg/kg) | $NO_3^-$ * (mg/kg) | Exchangeable K * (mg/kg) | Resin P (mg/kg) | Total C * (%) | Total N * (%) | Total P * (mg/kg, top 10cm; ignition and extraction) |
|---|---|---|---|---|---|---|---|---|---|---|---|---|
| 0 | 10.8 ± 1.3 | 36.1 ± 1.4 | 53.1 ± 1.1 | 5.4 ± 0.077 | 4.7 ± 0.075 | 12 ± 2.8 A | 0.8 ± 0.26 A | 120 ± 10 A | 0.86 ± 0.095 | 3.8 ± 0.079 A | 0.30 ± 0.094 AB | 293.0 ± 14 AB |
| 10 | 10.3 ± 1.0 | 33.7 ± 2.0 | 56.0 ± 2.4 | 5.2 ± 0.065 | 4.4 ± 0.072 | 5.0 ± 0.51 B | 0.12 ± 0.041 A | 110 ± 11 A | 0.55 ± 0.057 | 3.9 ± 0.10 AB | 0.28 ± 0.071 A | 255.4 ± 10.5 A |
| 30 | 13.0 ± 2.0 | 30.1 ± 1.7 | 56.9 ± 2.4 | 5.3 ± 0.087 | 4.4 ± 0.098 | 6.9 ± 0.63 B | 0.23 ± 0.083 A | 70 ± 7.9 B | 0.76 ± 0.16 | 4.2 ± 0.10 B | 0.32 ± 0.0099 B | 319.6 ± 15.7 B |
| 600 | 13.9 ± 1.3 | 12.4 ± 2.1 | 73.7 ± 2.2 | 5.5 ± 0.081 | 4.7 ± 0.073 | 1.4 ± 0.082 B | 3.3 ± 1.0 B | 110±12 AB | 0.45 ± 0.065 | 3.9 ± 0.21 AB | 0.37 ± 0.018 C | 303.4 ± 56.3 AB |

**Supplementary Table 2. Comparison of measurements of total phosphorus at Gigante in control plots. The table provides the study or dataset, total phosphorus measurements (mean +/- standard error), methods and notes.**

Supplementary Table 2 presents all values of total phosphorus recorded from Gigante for the four control plots. There is a substantial range in total phosphorus values which could be due to two sources of inconsistency. One is the way that phosphorus is measured (discussed below). The other is that total phosphorus is highly variable across the landscape in central Panama[6].

Total phosphorus was measured with four different methods. It was determined with ignition at 550 °C followed by acid digestion (1 M sulfuric acid) on four occasions with mean values for surface soils (0-10 or 0-15 cm) ranging from 305-359 mg kg$^{-1}$ (e.g., ref.[7-9]). We do not include the total phosphorus in the soil pit north because these soils are different from the soils on which are plots are located. Three total phosphorus values determined using other methods are much higher and are discounted: First, Koehler et al. (2009)[8] determined total phosphorus using pressure digestion with nitric acid followed by analysis with inductively coupled plasma-atomic emission spectrometry[10] (henceforth pressure digestion). Pressure digestion has an array of operational concerns and safety limitations that can cause errors[11,12]. Second, Mirabello et al. (2013)[9], and third, Yavitt et al. (2011)[1], summed Hedley fractions to estimate total phosphorus. This method is known to overestimate total phosphorus[13-18], although it has also been found to underestimate total phosphorus[6]. The situation is particularly problematic for highly weathered soils[13].

To obtain a gold standard estimate of total phosphorus (and metals) for Gigante, we extracted soils with concentrated nitric acid ($HNO_3$) at the Cornell Nutrient Analysis Laboratory (CNAL) at Cornell University and at the STRI soils laboratory. This method is based on the century-old extraction protocol for total recoverable metals in soil[19]. We believe the concentrated nitric acid extractions provide the best estimate of total phosphorus at Gigante because ignition and extraction is known to underestimate total phosphorus[17,18].

We scaled our surface total phosphorus to the deeper (~10-30 or ~15-30 cm depths) layers using quantified ratios (Figshare excel spreadsheet)[4], and explained here. For the 2018 data, we estimated total phosphorus for 0-30 cm by estimating total phosphorus for 15-30 cm from the value for 0-15 cm using the relationship in Yavitt et al 2011[1] (Table 1) and averaging the 0-15 and estimated 15-30 cm depth values. For the 2017 data, we used the ratio of deeper (~10-30 cm) to surface (~0-10 cm) to estimate the total phosphorus for 10-30 cm, and then took the depth-weighted mean of the 0-10 and 10-30 cm depth values. We then took the mean of the 2017 and 2018 0-30 cm data to get the total phosphorus at 0-30 cm for Gigante. We used this estimate to compare with values from across Amazonia obtained with the same nitric acid extraction (Quesada et al., 2010[20]; Supplementary Table 3).

**Supplementary Table 2. Continued**

| Location | Source of data (publication or dataset) | Year collected | Depth interval | Total P (mg / kg) mean ± 1 s.e.m. | Number of samples per plot | Total P (mg/kg) (values for control plots 6, 12, 26 and 36, respectively) | Method | Notes |
|---|---|---|---|---|---|---|---|---|
| Gigante control plots | Joe Yavitt (unpublished) | 2018 | 0-15 | 340.5 ± 25 | 1 | 360, 277, 393, 332 | nitric acid extraction | |
| | | | 15-30 | 283 | | | calculated | Calculated. Multiplied the 0-15 depth total P by the ratio of the 15-30 versus 0-15 depth total P for control plots in Table 1 of Yavitt et al (2011)[1]. (=341*(402/484)) |
| | | | 0-30 | 312 | | | calculated | Calculated as the mean of the 0-15 and 15-30 depths (341+283)/2 |
| Gigante control plots | Joe Yavitt (unpublished) | 2004 | 30-45 | 248 | | | ignition and acid extraction | Ignition (550C) and acid extraction |
| Gigante control plots | STRI soils laboratory (unpublished) | 2017 | 0-10 | 303 ± 56 | 1 | 177, 341, 439, 256 | nitric acid extraction | |
| | | | 10-30 | 240 | | | calculated | Calculated. Multiplied the 0-10 depth total P by the ratio of the deeper to surface soil total P in the Soil Pit South 2009 dataset (0-11 and 11-22 cm), STRI soils laboratory (unpublished) 2012 (0-5,5-10, 10-20) and Yavitt et al 2011[1] (0-15, 15-30 cm) data. (=303*(((282/359)+(230/((347+262)/2))+(402/484))/3)) |
| | | | 0-30 | 261 | | | calculated | Calculated as the depth-weighted mean of the 0-10 and 10-30 depths (=(303*10+240*20)/30) |

**Supplementary Table 2. Continued**

| Location | Source of data (publication or dataset) | Year collected | Depth interval | Total P (mg / kg) mean ± 1 s.e.m. | Number of samples per plot | Total P (mg/kg) (values for control plots 6, 12, 26 and 36, respectively) | Method | Notes |
|---|---|---|---|---|---|---|---|---|
| Gigante control plots | Mirabello et al 2013[9] - total P reported in text p.219 | 2006 | 0-15 | 340 | 1 | | ignition and acid extraction | ignition (550C) and acid extraction |
| | Mirabello et al 2013[9] - total P by adding the Hedley fractions, reported in text p.219 | | 0-15 | 680 | | | Hedley fractionation | The Hedley sequential fractionation is known to overestimate total P[17,18]. |
| Gigante control plots | Koehler et al 2009[8] Table 1 | 2006 | 0-5 | 550 ± 80 | 4 | | pressure digestion | Pressure digestion in concentrated $HNO_3$[10] followed by analysis with inductively coupled plasma-atomic emission spectrometer. |
| | | | 5-50 | 400 ± 70 | | | | |
| Gigante control plots | STRI soils laboratory (unpublished) | 2012 | 0-5 | 347 ± 44 | 1 | 254, 360, 460, 312 | ignition and acid extraction | Plot 26 has unusually high total P. Total phosphorus was determined by ignition (550°C, 1 h) and extraction in 1 M $H_2SO_4$ (16 h, 1:50 soil to solution ratio), with phosphate detection by automated neutralization and molybdate colorimetry on a Lachat Quickchem 8500 (Hach Ltd, Loveland, CO, USA). |
| | | | 5-10 | 262 ± 27 | | 194, 283, 319, 251 | | |
| | | | 10-20 | 230 ± 32 | | 156, 244, 308, 210 | | |
| | | | 20-50 | 175 ± 26 | | 121, 175, 245, 160 | | |
| | | | 50-100 | 124 ± 21 | | 77, 137, 175, 105 | | |

**Supplementary Table 2. Continued**

| Location | Source of data (publication or dataset) | Year collected | Depth interval | Total P (mg / kg) mean ± 1 s.e.m. | Number of samples per plot | Total P (mg/kg) (values for control plots 6, 12, 26 and 36, respectively) | Method | Notes |
|---|---|---|---|---|---|---|---|---|
| Soil pit north (Inceptisol) | Gigante Profile Descriptions Soil Classification.pdf; Turner & Benham, see ref.[4] | 2009 | 0-8 | 368 | 1 | | ignition and acid extraction | This pit contains soils that are different from the Gigante fertilization experiment. Total phosphorus was determined by ignition (550°C, 1 h) and extraction in 1 M $H_2SO_4$ (16 h, 1:50 soil to solution ratio), with phosphate detection by automated neutralization and molybdate colorimetry on a Lachat Quickchem 8500 (Hach Ltd, Loveland, CO, USA). |
| | | | 8-27 | 262 | | | | |
| | | | 27-80 | 236 | | | | |
| Soil pit south (Oxisol) | Gigante Profile Descriptions Soil Classification.pdf; Turner & Benham, see ref.[4] | 2009 | 0-11 | 359 | 1 | | ignition and acid extraction | This pit contains soils similar to those of the fertilization experiment. Total phosphorus was determined by ignition (550°C, 1 h) and extraction in 1 M $H_2SO_4$ (16 h, 1:50 soil to solution ratio), with phosphate detection by automated neutralization and molybdate colorimetry on a Lachat Quickchem 8500 (Hach Ltd, Loveland, CO, USA). |
| | | | 11-22 | 282 | | | | |
| | | | 22-50 | 191 | | | | |
| Gigante control plots | Yavitt et al., 2011[1], total P reported in the table 1 | 2011 | 0-15 | 484±33 | 1 | | Hedley fractions | The Hedley sequential fractionation is known to overestimate total P[17,18]. |
| | | | 15-30 | 402±34 | 1 | | | |

**Supplementary Table 3. Comparison of total phosphorus levels determined with concentrated nitric acid extraction for soils in our experiment and soils across the Amazon Basin.** Amazonian total phosphorus and proportional land area are from Table 4 in Quesada et al 2010[20] and Table 1 in Quesada et al 2011[21], respectively. We ordered soil types by total phosphorus and added the cumulative proportional area to highlight the comparison of our experiment with Amazonia. All total phosphorus was measured with nitric acid extraction.

| Soil type in forests of the Amazon Basin and how soils from our experiment compares | Mean total phosphorus (mg $kg^{-1}$) of soil type* | Proportion of forested land that contains this soil type across the Amazon Basin** | Cumulative proportion of forested land with this soil type across the Amazon Basin |
|---|---|---|---|
| Podzols | 29 | 0.019 | 0.019 |
| Arenosols | 36 | 0.027 | 0.046 |
| Ferralsols mean*** | 115 | 0.316 | 0.362 |
| Acrisols mean*** | 122 | 0.289 | 0.651 |
| Lixisols | 179 | 0.019 | 0.670 |
| Plinthisols mean*** | 226 | 0.087 | 0.757 |
| Agua Salud soil**** | 246 | | |
| Gigante soil**** | 286 | | |
| Alisols mean | 289 | 0.003 | 0.760 |
| Cambisols mean*** | 384 | 0.056 | 0.816 |
| Gleysols mean*** | 385 | 0.083 | 0.899 |
| Nitisols | 408 | 0.001 | 0.900 |
| Fluvisols | 669 | 0.025 | 0.925 |

* Values from Table 4 in Quesada et al 2010[20].
**Values from Table 1 in Quesada et al 2011[21].
*** For these soil types, we averaged multiple measurements from Table 4 in Quesada et al 2010[20].
**** We quantified total phosphorus for the following depths (in centimeters): 0-10 (2015-2016 data, all plots pre-fertilization Agua Salud, Supplementary Table 1), and 0-10 (STRI soils laboratory 2017 data, control plots Gigante) and 0-15 (Yavitt unpublished 2018 data, control plots Gigante) (Supplementary Table 2). We estimated the 0-30 cm depth values as depth-weighted means of surface values and values estimated for missing deeper depths. We estimated missing deeper values as the product of surface values and the ratio of deeper:surface values from nearby soil pits (Agua Salud; *see* Tang_etal_AguaSalud_Soil_pits.pdf in ref.[4]) and control plots (Gigante; *see* Tang_etal_Gigant_Soil_pits.pdf and Tang_etal_Gigantesoil_P.csv in ref.[4]and ref.[1]). Total phosphorus for Agua Salud is reported as the mean of the three forest ages. Total phosphorus for Gigante was taken as the mean of the 2017 and 2018 0-30 cm depth data (Supplementary Table 2).

**Supplementary Table 4. Statistical results of contrasts between treatments, including the effect of nitrogen and phosphorus on net change of aboveground biomass (AGB), biomass gain and biomass across forest ages.** This table reports the contrasts between plot scale data with versus without nitrogen (N; nitrogen and nitrogen plus phosphorus vs. control and phosphorus), and with versus without phosphorus (P; phosphorus and nitrogen plus phosphorus vs. control and nitrogen). Values are the p-values for each response variable. See Table 1 for the statistical results of the full model.

| Forest age | Variable | Relative to without N | Relative to without P |
|---|---|---|---|
| | | all N | all P |
| 0 years | Net change of AGB | **0.012** | |
| | Biomass gain | **0.00144** | |
| | Biomass loss | **0.00144** | **0.00144** |
| 10 years | Net change of AGB | **0.0039** | |
| | Biomass gain | **0.0132** | |
| | Biomass loss | 1 | 1 |
| 30 years | Net change of AGB | 1 | |
| | Biomass gain | 1 | |
| | Biomass loss | 1 | 1 |
| 600 years | Net change of AGB | 1 | |
| | Biomass gain | 1 | |
| | Biomass loss | 1 | 1 |

**Supplementary Table 5. Summary of studies that examine nutrient effects on stand-scale properties of wood carbon accumulation, including basal area increment and aboveground biomass increment in tropical forests. Forest type is recorded as reported by the authors.**

| Forest type | Treatment | Response | Response variable | Forest type (rainfall) | Location | Citation |
|---|---|---|---|---|---|---|
| Secondary (6 years) | C,N,P | N effect - 67% increase | Biomass increment | Moist lowland (1800 mm) | Brazil | Davidson et al 2004[22] |
| Secondary (30 years) | C,N,P | No effect | Biomass increment | Dry lowland (1800 mm) | Costa Rica | Waring et al 2019[23] |
| Secondary (24 years) | C,P | No effect | Biomass increment | Moist lowland (1760 mm) | Brazil | Markewitz et al 2012[24] |
| Mature | C,N | No effect | Biomass increment | Wet montane (5500 mm) | Panama | Adamek et al 2009[25] |
| Mature | C,N | No effect | Biomass increment | Wet lowland (3500 mm); Wet lower montane (4300 mm) | Puerto Rico | Cusack et al 2011[26] |
| Mature | C,N,P | No effect | Basal area increment | Moist montane (2200 mm) | Ecuador | Homeier et al 2012[27] |
| Mature | C,N,P | No effect | Basal area increment | Wet lowland (3464 mm) | Costa Rica | Alvarez-Clare et al 2013[28] |
| Mature | C,N,P | No effect | Biomass increment | Moist lowland (2400 mm) | Brazil | Cunha et al 2022[29] |
| Mature | C,N,P | P effect - 50% increase | Biomass increment | Moist montane (2500 mm) | Hawaii | Herbert & Fownes 1995[30] |
| Mature - primary succession* | C,N,P | N effect in early primary succession - 113% increase, no effect in late primary succession | Biomass increment | Moist montane (2500 mm) | Hawaii | Harrington et al 2001[31] |

***Note that the late primary succession site is the same as reported in Herbert & Fownes 1995[30], but more trees were measured (all trees versus 6 trees per plot) and the data were collected ~6-11 years after the initial results reported in Herbert & Fownes 1995[30].**

**Supplementary Information 2.**

**Estimate of additional annual carbon sequestration prevented in tropical forests due to nutrient limitation on recovering forests**

We estimated the potential for the prevention of additional carbon sequestration due to nutrient limitation in future reforestation across the tropics. We focus on new reforestation driven by a carbon price, since that would contribute additional carbon sequestered beyond business-as-usual reforestation. It also represents an incentivization that would allow tropical reforestation as a natural climate solution. Furthermore, we focus on this additional potential reforestation because it offers an opportunity to be strategic about reforestation efforts in terms of location and management practice, whereas business-as-usual reforestation may have less opportunity for strategic planning, especially location. Land abandonment and subsequent reforestation occurs for several reasons, including socio-economic, demographic and environmental factors[32-35]; maximizing carbon sequestration is usually not a driving factor for where or how reforestation is done. Any adjustment in business-as-usual reforestation to reduce nitrogen limitation and maximize carbon sequestration would further increase our carbon sequestration estimates.

Our estimates consider all types of reforestation – for example natural regeneration both managed and unmanaged, active tree planting, plantations and enrichment plantings within plantations or natural regeneration. Our estimates of additional carbon sequestration potential with the alleviation of nitrogen limitation are particularly relevant to natural regeneration because our forests are undergoing natural regeneration with limited management (e.g., fencing). Of all the reforestation types, managed or unmanaged natural regeneration reforestation approaches would likely be most feasible and beneficial in the future since natural regeneration is the primary method of reforestation enacted today[35], is more cost effective[33] and achieves higher ecological restoration success[36] than tree planting and other active reforestation practices. Despite this, natural regeneration is slated to be used in about one third of reforestation committed for the future under the Bonn Challenge[35,37], although much more area would benefit from and could support natural reforestation than is currently pledged[33,34,37].

To calculate how much carbon sequestration is prevented by nitrogen limitation, we extended the analysis by Busch et al. (2019)[38]. That analysis estimated the carbon sequestration potential of reforestation on previously forested lands in the tropics that are returned to forest owing to incentives from a hypothetical future carbon price. We estimate the maximum potential carbon sequestration given the alleviation of nutrient limitation to determine how much carbon sequestration is prevented by nutrient limitation. The benefit of the Busch et al.[38] model is that it provides an economically informed prediction of future additional carbon sequestration under different carbon prices as opposed to maximum potential reforestation given biophysical but not socioeconomic constraints (e.g., ref.[39]). The model considers characteristics of each site to project reforestation, including socioeconomic and political factors like agricultural revenue potential, protected status and distance from cities, and biophysical constraints like initial forest cover and slope, elevation, continent and biome. The analysis only allows reforestation on lands that have the potential for forest cover as determined by the existence of existing tree cover in the grid cell. Business as usual reforestation rates were projected based on reforestation rates between 2000 and 2010. The model assumes a mean carbon recovery pattern for regions, reforestation type (plantation versus non-plantation) and forest type (moist forest, dry forest and forest in grasslands), although carbon recovery rates can vary widely across these different categories[40].

We focus on scenarios where the carbon price is \$100 $tCO_2^{-1}$ because this price is well below mean estimates of the social cost of carbon[41] and is on the right order of magnitude for mitigation pathways consistent with Paris Agreement temperature targets[41, 42]. \$100 $tCO_2^{-1}$ is also considered the maximum carbon price for reforestation[43]. Considering a lower cost of carbon – such as one more similar to today – would result in less additional carbon sequestered. At \$100 $tCO_2^{-1}$, Busch et al.[38] project a $CO_2$ sequestration rate due to reforestation of up to 1.6 Gt $CO_2$ $year^{-1}$ (the maximum sequestration rate that is projected to occur between 2040-2050) between 2020 and 2050[38]. This is on the lower end of the range projected by other studies (as discussed in Fuss et al. 2018[43]), and thus we consider our estimates conservative for the carbon price.

We use the projected additional reforestation at \$100 $tCO_2^{-1}$ and our experimental results of the effect of nutrients on forest carbon accumulation to generate a carbon multiplier for reforestation alleviated of nitrogen limitation. This multiplier represents the additional carbon sequestered due to alleviation of nutrient limitation based on our experimental data. We apply this multiplier to tropical forests globally, as described below.

First, to calculate the nutrient carbon multiplier, we estimated the additional carbon stored when nutrients are not limiting compared to when nutrients are limiting from our net biomass change data. We do this for forests between 0 and 10 years (mean age of 5 years, assuming new forests are being added each year at the same rate), 10 and 20 years and 20 and 30 years. We assume that forests sequester 95% more carbon in the first four years of recovery, and then that they have a linear decline in additional carbon stored between 4 (95%) and 14 (48%) years and between 14 and 30 (0%) years. Years 4, 14 and 30 are the ages of our 0-, 10- and ~30- year old forests at the end of the period of data collection. We also assume that additional biomass was equivalent to additional carbon stored. Thus, we estimate that forests between 0 to 10 years sequester a mean of 87% more biomass carbon without nutrient limitation than forests with nutrient limitation. We estimate that forests between 10 and 20 years store 46% more carbon and forests between 20 and 30 years sequester 15% more carbon.

Second, we determine how much additional carbon that would be sequestered (i.e., abatement) per year when the carbon price is \$100 $tCO_2^{-1}$. Data was extracted from figure 1 of Busch et al. 2019[38] which contains carbon that is projected to be sequestered for the business-as-usual and \$100 $tCO_2^{-1}$ scenarios. Data extraction was done using webplotdigitizer. The difference between business-as-usual and \$100 $tCO_2^{-1}$ scenario is the additional carbon sequestered. We did this for each decade between 2020 and 2050. There is an additional 0.54, 1.14 and 1.6 Gt $CO_2$ $year^{-1}$ stored because of the carbon price in 2020-2030, 2030-2040 and 2040-2050, respectively.

Third, to determine how the area of different forest ages changes over time, we track the land area that has forests 0-10, 10-20 and 20-30 years old for each time period (2020-2030, 2030-2040, 2040-2050) by extracting data for the land reforested in the business-as-usual and the \$100 $tCO_2$ scenarios in Supplementary Figure 6 of Busch et al., 2019[38] with webplotdigitizer. The difference is the additional land area reforested because of the carbon price. Reforestation is projected to occur on 53.8, 61.3 and 69.3 Mha $year^{-1}$ in periods 2020-2030, 2030-2040 and 2040-2050, respectively.

Fourth, we use these numbers to determine the fraction of reforested area in each forest age class at each time period when considering the amount of land area in each age class and the total land area reforested. From 2020-2030, 100% of projected reforested areas would have forests that are 0-10 years old. From 2030-2040, 53% of projected reforested

areas would have forests that are 0-10 years old and 47% would have forests that are 10-20 years old. From 2040-2050, 38% of projected reforested areas would have forests that are 0-10 years old, 33% would have forests that are 10-20 years old and 29% would have forests that are 20-30 years old.

Fifth, we took the product of the carbon multiplier for each forest age, the fraction of reforested land area in each forest age and the additional carbon sequestered per year for each time period to estimate additional carbon sequestration due to the alleviation of nutrient limitation. We found that the additional carbon sequestered from reforestation without nutrient limitation is up to 0.84 Gt $CO_2$ year$^{-1}$. In 2020-2050, additional reforestation without nutrient limitation could sequester up to 0.47 Gt $CO_2$ year$^{-1}$; in 2030-2040, this number increases to 0.77 Gt $CO_2$ year$^{-1}$; and, in 2040-2050, this number is 0.84 Gt $CO_2$ year$^{-1}$. On average across the three time periods, reforested areas that are not nutrient limited sequester an additional 0.69 Gt $CO_2$ year$^{-1}$.

Importantly, increasing the carbon sequestration potential per hectare reforested means that reforestation becomes a more attractive carbon mitigation option at lower carbon prices, and increasing the land area that would be economically attractive for reforestation at \$100 t$CO_2^{-1}$.

Our projections assume that there is no cost to implementing the enhanced reforestation practices. First, this is reasonable for the strategy of planting nitrogen-fixing trees since these species can be substituted for non-fixing species on a one-to-one basis in active restoration. There would be a cost to planting nitrogen-fixing trees in passive regeneration. Second, on a first approximation, there would be no intrinsic additional cost to prioritizing reforestation on fertile soils (made available under the carbon price) since tree planting costs would be similar per land area and unmanaged natural regeneration could occur anywhere at the same cost. In contrast, managed natural regeneration could vary in cost across different areas because of access to roads and municipalities where workers live, etc. More fertile lands may nonetheless retain more relative value for high yield agriculture, which should be considered in future, more detailed analyses. Third, reforesting on lands that have higher nitrogen deposition would also not intrinsically be more costly than reforesting on lands with lower nitrogen deposition because nitrogen deposition is widespread regionally[44,45], although there may be a lower cost of fertilizer use for crops on these lands, which should also be considered in future analyses.

If the cost of alleviating nutrient limitation were significant, it would offset the benefit of the carbon multiplier. Using fertilizer in reforestation efforts – which we do not advocate due to the potential negative externalities that would require substantial further study – would come with potentially large additional costs, including for fertilizer and labour. At the current market price of \$300 ton$^{-1}$ urea, urea at 46.7% nitrogen and a fertilization rate of 125 kg nitrogen ha$^{-1}$ year$^{-1}$, we approximate that fertilizer would cost \$80 ha$^{-1}$ year$^{-1}$. In Panama, fertilizer can be spread by hand in our experiment at a rate of 2 people ha$^{-1}$ day$^{-1}$, assuming wages are at minimum \$20 person$^{-1}$ day$^{-1}$ (but would likely cost more to run a fertilization program) and forests must be fertilized four times per year over the wet season. This results in a cost of approximately \$160 ha$^{-1}$ year$^{-1}$ for labour, or \$247 ha$^{-1}$ year$^{-1}$ when including the cost of fertilizer. Considering that our fertilized forests sequester *an additional* ~4 t$CO_2$ ha$^{-1}$ year$^{-1}$ in the first ten years of reforestation, the cost to using fertilizer erodes the net financial benefit of nutrient enhancement at \$100 t$CO_2^{-1}$ by 60%. A cost of carbon below \$60 t$CO_2^{-1}$ would provide no incentive for nutrient enhancement through fertilizer application. External costs can further decrease the economic benefit of fertilizer application to enhance sequestration from reforestation. Costs not considered here include additional costs of

fertilizing beyond labour and urea, high rates of nitrous oxide emissions which has a global warming potential of 298 relative to $CO_2$[46], emissions related to the production of fertilizer[47], eutrophication of waterways due to nitrogen runoff, decline in soil pH[48] and the loss of biodiversity[49].

## References

1. Yavitt, J.B. et al. Soil fertility and fine root dynamics in response to 4 years of nutrient (N, P, K) fertilization in a lowland tropical moist forest, Panama. *Austral Ecol.* **36**, 433–445 (2011).

2. Wright, S.J. et al. Insights from a long-term, factorial nitrogen, phosphorus, and potassium addition experiment conducted in a mature lowland tropical forest in the Barro Colorado Nature Monument. Pages 539-547. In: *Muller-Landau HC, Wright SJ (eds) The First 100 Years of Research on Barro Colorado: Plant and Ecosystem Science*. Smithsonian Institution Scholarly Press, Washington, DC (2024).

3. Turner, B.L. et al. Seasonal changes and treatment effects on soil inorganic nutrients following a decade of fertilization in a lowland tropical forest. *Soil Sci. Soc. Am. J.* **77**: 1357–1369 (2013).

4. Tang, W. et al. Data and code associated with: Tang et al. Tropical forest carbon sequestration accelerated by nitrogen. Cary Institute. Dataset. https://doi.org/10.25390/caryinstitute.25892776.v1. (2025).

5. R. Core Team, R: A language and environment for statistical computing. *R Foundation for Statistical Computing*. (Vienna, Austria 2021).

6. Dieter, D., Elsenbeer, H. & Turner, B. L. Phosphorus fractionation in lowland tropical rainforest soils in central Panama. *Catena* **82**, 118–125 (2010).

7. Wright, S.J. et al. Potassium, phosphorus, or nitrogen limit root allocation, tree growth, or litter production in a lowland tropical forest. *Ecology* **92**, 1616–1625 (2011).

8. Koehler, B. et al. Immediate and long-term nitrogen oxide emissions from tropical forest soils exposed to elevated nitrogen input. *Glob. Change Biol.* **15**, 2049–2066 (2009).

9. Mirabello, M. et al. Soil phosphorus responses to chronic nutrient fertilisation and seasonal drought in a humid lowland forest, Panama. *Soil Res.* **51**, 215–221 (2013).

10. König, N., Fortmann, H. & Lüter, K. Probenvorbereitungs-, Untersuchungs- und Elementbestimmungs-Methoden des Umweltanalytik-Labors der Niedersächsischen Forstlichen Versuchsanstalt. 2. Ergänzung: 1999-2008. Teil 4: Elementbestimmungsmethodenp-Zn und Sammelanhänge. Berichte des Forschungszentrums Waldökosysteme **Reihe B**, (2009).

11. Gasparatos, D. & Haidouti, C. A comparison of wet oxidation methods for determination of total phosphorus in soils. *J. Plant Nutr. Soil Sci.* **164**, 435-439 (2001).

12. Matusiewicz, H. Sample preparation for inorganic trace element analysis. *Phys. Sci. Rev.* **2**, p.20178001 (2017).

13. Williams, J.D.H. et al. A comparison of methods for the determination of soil organic phosphorus. *Soil Sci.* **110,** 13–18 (1970).

14. Oniani, O.G., Chater, M. & Mattingly, G.E.G. Some effects of fertilizers and farmyard manure on the organic phosphorus in soils. *J. Soil Sci.* **24,** 1–9 (1973).

15. Do Nascimento, C.A. et al. Phosphorus concentrations in sequentially fractionated soil samples as affected by digestion methods. *Sci. Rep.* **5**, 17967 (2015).

16. Guggenberger, G. et al. Land-use and fertilization effects on P forms in two European soils: resin extraction and 31P-NMR analysis. *Eur. J. Soil Sci.* **47,** 605–614 (1996).

17. Gu, C. et al. Quantifying uncertainties in sequential chemical extraction of soil phosphorus using XANES spectroscopy. *Environ. Sci. Technol.* **54**, 2257–2267 (2020).

18. Turner, B. L. et al. Extraction of soil organic phosphorus. *Talanta* **66**, 294–306 (2005).

19. Church, C., Spargo, J., & Fishel, S. Strong acid extraction methods for "total phosphorus" in soils: EPA method 3050B and EPA method 3051. *Agric. Environ. Lett.* **2**, 160037 (2017).

20. Quesada, C.A. et al. Variations in chemical and physical properties of Amazon forest soils in relation to their genesis. *Biogeosciences* **7**, 1515–1541 (2010).

21. Quesada, C. et al. Soils of Amazonia with particular reference to the RAINFOR sites. *Biogeosciences* **8**, 1415–1440 (2011).

22. Davidson, E.A. et al. Nitrogen and phosphorus limitation of biomass growth in a tropical secondary forest. *Ecol. Appl.* **14**, 150–163 (2004).

23. Waring, B.G. et al. Plant community responses to stand-level nutrient fertilization in a secondary tropical dry forest. *Ecology* **100**, e02691 (2019).

24. Markewitz, D. et al. Soil and tree response to P fertilization in a secondary tropical forest supported by an Oxisol. *Biol. Fertil. Soils* **48**, 665–678 (2012).

25. Adamek, M., Corre, M. D. & Hölscher, D. Early effect of elevated nitrogen input on above-ground net primary production of a lower montane rain forest, Panama. *J. Trop. Ecol.* **25**, 637–647 (2009).

26. Cusack, D. F. et al. Effects of nitrogen additions on above- and belowground carbon dynamics in two tropical forests. *Biogeochemistry* **104**, 203–225 (2011).

27. Homeier, J. et al. Tropical Andean Forests Are Highly Susceptible to Nutrient Inputs—Rapid Effects of Experimental N and P Addition to an Ecuadorian Montane Forest. *PloS ONE* **7**, e47128 (2012).

28. Alvarez-Clare, S., Mack, M.C. & Brooks, M. A direct test of nitrogen and phosphorus limitation to net primary productivity in a lowland tropical wet forest. *Ecology* **94**, 1540–1551 (2013).

29. Cunha, H.F.V. et al. Direct evidence for phosphorus limitation on Amazon forest productivity. *Nature* **608**, 558–562 (2022).

30. Herbert, D.A. & Fownes, J.H. Phosphorus limitation of forest leaf area and net primary production on a highly weathered soil. *Biogeochemistry* **29**, 223–235 (1995).

31. Harrington, R. A., Fownes, J. H. & Vitousek, P. M. Production and resource use efficiencies in N-and P-limited tropical forests: a comparison of responses to long-term fertilization. *Ecosystems* **4**, 646–657 (2001).

32. Lambin, E.F., Geist, H.J. & Lepers, E. Dynamics of land-use and land-cover change in tropical regions. *Annu. Rev. Environ. Resour.* **28**, 205–241 (2003).

33. Chazdon, R.L. & Guariguata, M.R. Natural regeneration as a tool for large-scale forest restoration in the tropics: prospects and challenges. *Biotropica* **48**, 716-730 (2016).

34. Williams, B.A. et al. Global potential for natural regeneration in deforested tropical regions. *Nature* **636**, 131–137 (2024).

35. Gao, X. et al. The importance of distinguishing between natural and managed tree cover gains in the moist tropics. *Nat. Commun.* **16**, 6092 (2025).

36. Crouzeilles, R. et al. Ecological restoration success is higher for natural regeneration than for active restoration in tropical forests. *Sci. Adv.* **3**, e1701345 (2017).

37. Lewis, S.L. et al. Regenerate natural forests to store carbon. *Nature* **568**, 25–28 (2019).

38. Busch, J. et al. Potential for low-cost carbon dioxide removal through tropical reforestation. *Nat. Clim. Change* **9**, 463–466 (2019).

39. Griscom, B.W. et al. Natural climate solutions. *Proc. Natl. Acad. Sci.* **114**, 11645–11650 (2017).

40. Cook-Patton, S.C. et al. Mapping carbon accumulation potential from global natural forest regrowth. *Nature* **585**, 545–550 (2020).

41. Rennert, K. et al. Comprehensive evidence implies a higher social cost of $CO_2$. *Nature* **610**, 687–692 (2022).

42. Gambhir, A. et al. Adjusting 1.5 degree C climate change mitigation pathways in light of adverse new information. *Nat. Commun.* **14**, 5117 (2023).

43. Fuss, S. et al. Negative emissions—Part 2: Costs, potentials and side effects. *Environ. Res. Lett.* **13**, 063002 (2018).

44. Lamarque, J.F. et al. Multi-model mean nitrogen and sulfur deposition from the Atmospheric Chemistry and Climate Model Intercomparison Project (ACCMIP): evaluation of historical and projected future changes. *Atmo. Chem. Phys.* **13**, 7997–8018 (2013).

45. Gurmesa, G.A. et al. Retention of deposited ammonium and nitrate and its impact on the global forest carbon sink. *Nat. Commun.* **13,** 880 (2022).

46. Hall, S.J. & Matson, P.A. Nitrogen oxide emissions after nitrogen additions in tropical forests. *Nature* **400**, 152–155 (1999).

47. Menegat, S., Ledo, A. & Tirado, R. Greenhouse gas emissions from global production and use of nitrogen synthetic fertilisers in agriculture. *Sci. Rep.* **12**, 14490 (2022).

48. Matson, P.A. et al. The globalization of N deposition: ecosystem consequences in tropical environments. *Biogeochemistry* **46**, 67–83 (1999).

49. Lu, X. et al. Effects of experimental nitrogen additions on plant diversity in an old-growth tropical forest. *Glob. Change Biol.* **16**, 2688–2700 (2010).
